# Supplementary material for: End Sequence Analysis Toolkit (ESAT) expands the extractable information from single-cell RNA-seq data
Source: Genome Res. 2016 Oct;26(10):1397–410. doi: 10.1101/gr.207902.116 (PMC5052061; doi:10.1101/gr.207902.116)
Supplement: Supplemental Material [file supp_gr.207902.116_Supp_Materials.tar.gz › ESAT-esat_v0.1/doc/html/classbroad_1_1core_1_1motif_1_1_position_weight_column.html]

ESAT: broad.core.motif.PositionWeightColumn Class Reference


ESAT


- Main Page
- Related Pages
- Packages
- Classes
- Files

- Class List
- Class Index
- Class Hierarchy
- Class Members

All Classes Namespaces Files Functions Variables Enumerator Pages

- broad
- core
- motif
- PositionWeightColumn

Public Member Functions |
Static Public Attributes |
Protected Member Functions |
List of all members

broad.core.motif.PositionWeightColumn Class Reference

|  |  |
| --- | --- |
| Public Member Functions | |
|  | PositionWeightColumn () |
|  | |
|  | PositionWeightColumn (String[] rawData) |
|  | |
|  | PositionWeightColumn (int[] n) |
|  | |
|  | PositionWeightColumn (int nA, int nC, int nG, int nT) |
|  | |
|  | PositionWeightColumn (double[] freq) |
|  | |
| char | getBaseIUB () |
|  | |
| void | resetProbWithNullPriorProb () |
|  | |
| void | setNum (int[] n) |
|  | |
| void | setNum (int nA, int nC, int nG, int nT) |
|  | |
| PositionWeightColumn | getComplement () |
|  | |
| void | init (int alphabetSize) |
|  | |
| void | init () |
|  | |
| double | getLogRatio (char ch) |
|  | |
| double | getLogRatioWeightedByNegEntropy (char ch) |
|  | |
| double | getLogProb (char ch) |
|  | |
| double | getLogProb (int baseIdx) |
|  | |
| double | getLogRatio (char i, boolean isNumSeq) |
|  | |
| double | getLogRatioWeightedByNegEntropy (char i, boolean isNumSeq) |
|  | |
| void | computeProb () |
|  | |
| void | printProb () |
|  | |
| void | print () |
|  | |
| char | toConsensus () |
|  | |
| double | getInformationContent () |
|  | |
| void | flatWeight (double infoTh) |
|  | |
| void | resetToUniformProb () |
|  | |
| double | getWeight (int letterIndex) |
|  | |
| void | write (BufferedWriter bw, NumberFormat formatter) throws IOException |
|  | |
| String | toString () |
|  | |
| double | getProbability (int baseIndex) |
|  | |
| int | getAlphabetSize () |
|  | |
| void | addPseudoCounts () |
|  | |
| double | kullbackLeiber (PositionWeightColumn pwc) |
|  | |

|  |  |
| --- | --- |
| Static Public Attributes | |
| static final int | DEFAULT\_ALPHABET\_SIZE = 4 |
|  | |

|  |  |
| --- | --- |
| Protected Member Functions | |
| void | setFromCounts (int[] n) |
|  | |

## Constructor & Destructor Documentation

|  |  |  |  |  |
| --- | --- | --- | --- | --- |
| broad.core.motif.PositionWeightColumn.PositionWeightColumn | ( |  | ) |  |

Here is the call graph for this function:

Here is the caller graph for this function:

|  |  |  |  |  |  |
| --- | --- | --- | --- | --- | --- |
| broad.core.motif.PositionWeightColumn.PositionWeightColumn | ( | String[] | *rawData* | ) |  |

Here is the call graph for this function:

|  |  |  |  |  |  |
| --- | --- | --- | --- | --- | --- |
| broad.core.motif.PositionWeightColumn.PositionWeightColumn | ( | int[] | *n* | ) |  |

Here is the call graph for this function:

|  |  |  |  |
| --- | --- | --- | --- |
| broad.core.motif.PositionWeightColumn.PositionWeightColumn | ( | int | *nA*, |
|  |  | int | *nC*, |
|  |  | int | *nG*, |
|  |  | int | *nT* |
|  | ) |  |  |

Here is the call graph for this function:

|  |  |  |  |  |  |
| --- | --- | --- | --- | --- | --- |
| broad.core.motif.PositionWeightColumn.PositionWeightColumn | ( | double[] | *freq* | ) |  |

Here is the call graph for this function:

## Member Function Documentation

|  |  |  |  |  |
| --- | --- | --- | --- | --- |
| void broad.core.motif.PositionWeightColumn.addPseudoCounts | ( |  | ) |  |

Here is the call graph for this function:

|  |  |  |  |  |
| --- | --- | --- | --- | --- |
| void broad.core.motif.PositionWeightColumn.computeProb | ( |  | ) |  |

Here is the call graph for this function:

Here is the caller graph for this function:

|  |  |  |  |  |  |
| --- | --- | --- | --- | --- | --- |
| void broad.core.motif.PositionWeightColumn.flatWeight | ( | double | *infoTh* | ) |  |

Here is the call graph for this function:

|  |  |  |  |  |
| --- | --- | --- | --- | --- |
| int broad.core.motif.PositionWeightColumn.getAlphabetSize | ( |  | ) |  |

Here is the caller graph for this function:

|  |  |  |  |  |
| --- | --- | --- | --- | --- |
| char broad.core.motif.PositionWeightColumn.getBaseIUB | ( |  | ) |  |

|  |  |  |  |  |
| --- | --- | --- | --- | --- |
| PositionWeightColumn broad.core.motif.PositionWeightColumn.getComplement | ( |  | ) |  |

Here is the call graph for this function:

|  |  |  |  |  |
| --- | --- | --- | --- | --- |
| double broad.core.motif.PositionWeightColumn.getInformationContent | ( |  | ) |  |

Here is the call graph for this function:

Here is the caller graph for this function:

|  |  |  |  |  |  |
| --- | --- | --- | --- | --- | --- |
| double broad.core.motif.PositionWeightColumn.getLogProb | ( | char | *ch* | ) |  |

Here is the caller graph for this function:

|  |  |  |  |  |  |
| --- | --- | --- | --- | --- | --- |
| double broad.core.motif.PositionWeightColumn.getLogProb | ( | int | *baseIdx* | ) |  |

|  |  |  |  |  |  |
| --- | --- | --- | --- | --- | --- |
| double broad.core.motif.PositionWeightColumn.getLogRatio | ( | char | *ch* | ) |  |

Here is the call graph for this function:

|  |  |  |  |
| --- | --- | --- | --- |
| double broad.core.motif.PositionWeightColumn.getLogRatio | ( | char | *i*, |
|  |  | boolean | *isNumSeq* |
|  | ) |  |  |

Here is the call graph for this function:

|  |  |  |  |  |  |
| --- | --- | --- | --- | --- | --- |
| double broad.core.motif.PositionWeightColumn.getLogRatioWeightedByNegEntropy | ( | char | *ch* | ) |  |

Here is the call graph for this function:

|  |  |  |  |
| --- | --- | --- | --- |
| double broad.core.motif.PositionWeightColumn.getLogRatioWeightedByNegEntropy | ( | char | *i*, |
|  |  | boolean | *isNumSeq* |
|  | ) |  |  |

Here is the call graph for this function:

|  |  |  |  |  |  |
| --- | --- | --- | --- | --- | --- |
| double broad.core.motif.PositionWeightColumn.getProbability | ( | int | *baseIndex* | ) |  |

Here is the caller graph for this function:

|  |  |  |  |  |  |
| --- | --- | --- | --- | --- | --- |
| double broad.core.motif.PositionWeightColumn.getWeight | ( | int | *letterIndex* | ) |  |

Here is the caller graph for this function:

|  |  |  |  |  |  |
| --- | --- | --- | --- | --- | --- |
| void broad.core.motif.PositionWeightColumn.init | ( | int | *alphabetSize* | ) |  |

|  |  |  |  |  |
| --- | --- | --- | --- | --- |
| void broad.core.motif.PositionWeightColumn.init | ( |  | ) |  |

Here is the caller graph for this function:

|  |  |  |  |  |  |
| --- | --- | --- | --- | --- | --- |
| double broad.core.motif.PositionWeightColumn.kullbackLeiber | ( | PositionWeightColumn | *pwc* | ) |  |

Here is the call graph for this function:

|  |  |  |  |  |
| --- | --- | --- | --- | --- |
| void broad.core.motif.PositionWeightColumn.print | ( |  | ) |  |

Here is the call graph for this function:

|  |  |  |  |  |
| --- | --- | --- | --- | --- |
| void broad.core.motif.PositionWeightColumn.printProb | ( |  | ) |  |

Here is the call graph for this function:

|  |  |  |  |  |
| --- | --- | --- | --- | --- |
| void broad.core.motif.PositionWeightColumn.resetProbWithNullPriorProb | ( |  | ) |  |

Here is the call graph for this function:

|  |  |  |  |  |
| --- | --- | --- | --- | --- |
| void broad.core.motif.PositionWeightColumn.resetToUniformProb | ( |  | ) |  |

Here is the call graph for this function:

Here is the caller graph for this function:

|  |  |  |  |  |  |
| --- | --- | --- | --- | --- | --- |
| void broad.core.motif.PositionWeightColumn.setFromCounts | ( | int[] | *n* | ) |  |

protected

Here is the call graph for this function:

Here is the caller graph for this function:

|  |  |  |  |  |  |
| --- | --- | --- | --- | --- | --- |
| void broad.core.motif.PositionWeightColumn.setNum | ( | int[] | *n* | ) |  |

Here is the call graph for this function:

Here is the caller graph for this function:

|  |  |  |  |
| --- | --- | --- | --- |
| void broad.core.motif.PositionWeightColumn.setNum | ( | int | *nA*, |
|  |  | int | *nC*, |
|  |  | int | *nG*, |
|  |  | int | *nT* |
|  | ) |  |  |

Here is the call graph for this function:

|  |  |  |  |  |
| --- | --- | --- | --- | --- |
| char broad.core.motif.PositionWeightColumn.toConsensus | ( |  | ) |  |

Here is the caller graph for this function:

|  |  |  |  |  |
| --- | --- | --- | --- | --- |
| String broad.core.motif.PositionWeightColumn.toString | ( |  | ) |  |

Here is the call graph for this function:

|  |  |  |  |
| --- | --- | --- | --- |
| void broad.core.motif.PositionWeightColumn.write | ( | BufferedWriter | *bw*, |
|  |  | NumberFormat | *formatter* |
|  | ) |  | throws IOException |

Here is the call graph for this function:

## Member Data Documentation

final int broad.core.motif.PositionWeightColumn.DEFAULT\_ALPHABET\_SIZE = 4

static

---

The documentation for this class was generated from the following file:

- java/broad/core/motif/PositionWeightColumn.java


---

Generated on Fri Aug 15 2014 15:10:54 for ESAT by  

 1.8.7
